# Saliva and blood miRNAs as complementary biomarkers for esophageal cancer detection

**DOI:** 10.3389/fonc.2025.1642705

**Published:** 2026-01-05

**Authors:** Mary Kathryn Maxwell, Taichiro Nonaka

**Affiliations:** 1 School of Medicine, Louisiana State University Health Shreveport, Shreveport, LA, United States; 2 Department of Cellular Biology and Anatomy, Louisiana State University Health Sciences Center, Shreveport, LA, United States; 3 Feist-Weiller Cancer Center, Louisiana State University Health Shreveport, Shreveport, LA, United States

**Keywords:** esophageal cancer, microRNA, circulating biomarker, liquid biopsy, saliva diagnostics

## Abstract

**Objective:**

Esophageal cancer is a highly aggressive malignancy with poor survival rates due to late-stage diagnosis. Early detection is crucial for improving prognosis, yet current diagnostic methods, such as endoscopy, are invasive and impractical for routine screening. Circulating microRNAs (miRNAs) have emerged as promising non-invasive biomarkers for cancer detection through liquid biopsy approaches. This meta-analysis aims to evaluate the diagnostic performance of circulating miRNAs in blood and saliva for esophageal cancer detection.

**Methods:**

A systematic literature search was conducted in PubMed, Web of Science, and Scopus to identify relevant studies published between 2004 and 2024. Eligible studies included those that evaluated miRNA expression in plasma, serum, or saliva of esophageal cancer patients. The analysis assessed the diagnostic accuracy of circulating miRNAs in three distinct categories: combined blood- and saliva-derived miRNAs, blood-derived miRNAs, and saliva-derived miRNAs. Pooled sensitivity, specificity, summary receiver operating characteristic (SROC) curves, diagnostic likelihood ratios, and diagnostic odds ratios were evaluated to determine the reliability of these biomarkers.

**Results:**

A total of 27 articles encompassing 47 sub-studies with 2,396 patients were included in this meta-analysis. The pooled sensitivity of combined blood- and saliva-derived miRNAs for esophageal cancer diagnosis was 0.79 (95% CI: 0.76–0.82), with a specificity of 0.77 (95% CI: 0.72–0.80). Blood-derived miRNAs alone demonstrated a sensitivity of 0.77 and specificity of 0.79, while saliva-derived miRNAs alone exhibited higher sensitivity of 0.88 but lower specificity of 0.60. The area under the SROC curve (AUC) was 0.85 for both combined and blood-derived miRNAs, and 0.83 for saliva-derived miRNAs, demonstrating strong overall diagnostic accuracy. Diagnostic odds ratios further supported the clinical utility of miRNA biomarkers. Deeks’ funnel plot asymmetry test revealed no significant publication bias.

**Conclusion:**

Circulating miRNAs show strong potential as liquid biopsy biomarkers for the non-invasive diagnosis of esophageal cancer. While saliva-derived miRNAs exhibit higher sensitivity, blood-derived miRNAs provide greater specificity. These findings indicate the need for further validation and standardization of miRNA-based assays to facilitate their integration into clinical practice for early detection of esophageal cancer.

## 1 Introduction

Esophageal cancer is a malignant disease with a high global incidence and mortality rate (1). It ranks among the leading causes of cancer-related deaths worldwide, with a particularly high prevalence in Asia, parts of Africa, and Eastern Europe (2). The highest incidence rates are observed in China, Iran, and some sub-Saharan African countries, where dietary habits, environmental factors, and genetic predisposition play significant roles (3). According to recent global cancer statistics, more than 600,000 new cases of esophageal cancer are diagnosed annually (3). The mortality rate remains high, with an estimated 544,000 deaths reported in 2020, accounting for an overall case-fatality rate of approximately 90% (3). This high mortality is primarily due to late-stage detection and the limited effectiveness of current treatment options. In the United States, the five-year survival rate is 22%, highlighting the urgent need for improved early detection and therapeutic strategies (4). The disease predominantly affects males, with an estimated male-to-female ratio of approximately 3.75:1 in 2025, and it is more common in individuals over the age of 60 (4, 5). The disease is often diagnosed at an advanced stage because of the absence of early symptoms, making early detection crucial for improving survival rates.

Esophageal cancer is often asymptomatic in its early stages, which significantly hinders early diagnosis (6). The initial signs are usually subtle and may include mild discomfort while swallowing. As the tumor progresses, patients commonly experience dysphagia (difficulty swallowing), retrosternal pain, and unexplained weight loss (7). More advanced disease stages present with persistent cough, hoarseness, odynophagia (pain while swallowing), chest or back pain, and regurgitation. These symptoms often overlap with benign gastrointestinal or respiratory conditions, which can lead to misdiagnosis and further delays in intervention (8).

The two primary histological types of esophageal cancer include squamous cell carcinoma (SCC), which is more common in Asian populations, and adenocarcinoma, which is frequently associated with gastroesophageal reflux disease (GERD) and Barrett’s esophagus (9). The development of esophageal cancer is strongly associated with environmental and lifestyle factors, particularly smoking and alcohol consumption (10). SCC, the predominant histological type in Asian populations, is significantly linked to tobacco and alcohol use. Acetaldehyde, a metabolic byproduct of alcohol, is a known carcinogen, and individuals with genetic variations that impair acetaldehyde metabolism are at a higher risk (11). Moreover, the combination of smoking and alcohol use has a synergistic effect, significantly elevating cancer risk (12).

In addition to SCC, adenocarcinoma of the esophagus is primarily associated with GERD (13, 14). Chronic inflammation due to acid reflux can lead to Barrett’s esophagus, a condition where the normal squamous epithelium of the esophagus is replaced by metaplastic columnar cells. This metaplasia increases the risk of malignant transformation. Other contributing factors to esophageal cancer include obesity, dietary deficiencies, and prolonged consumption of hot beverages, which can cause thermal injury to the esophageal mucosa (13, 15, 16). Esophageal cancer is also known to metastasize rapidly due to its anatomical proximity to vital organs such as the lungs, heart, and major blood vessels (17, 18). Even early-stage esophageal cancer has a high tendency for lymph node metastasis, making it one of the most aggressive malignancies in the gastrointestinal tract (19).

Currently, the primary screening and diagnostic methods for esophageal cancer include upper gastrointestinal endoscopy and barium swallow radiography (20, 21). However, these methods are invasive, costly, and often associated with patient discomfort, leading to poor compliance. While endoscopy remains the gold standard for detecting esophageal lesions, its routine application for early-stage screening in asymptomatic populations is impractical (22). There is an urgent need for non-invasive, patient-friendly diagnostic methods that facilitate early detection and improve survival outcomes.

In recent years, liquid biopsy has gained attention as a promising non-invasive approach for cancer diagnosis (23). Among the circulating biomarkers identified in liquid biopsies, circulating microRNAs (miRNAs) have emerged as potential diagnostic tools (24). miRNAs are short, non-coding RNAs that regulate gene expression and play a critical role in cancer development and progression (25). Cancer cells, including those of esophageal cancer, release exosomal miRNAs into the bloodstream, making them accessible biomarkers for disease detection (26). Exosomal miRNAs exhibit remarkable stability in circulation due to their encapsulation within lipid vesicles, which protect them from enzymatic degradation (27). These miRNAs reflect the molecular characteristics of tumor cells and may provide a comprehensive representation of cancer status (28). Consequently, circulating miRNAs have gained significant interest as potential biomarkers for liquid biopsy-based cancer diagnostics (29). Additionally, studies have demonstrated that specific miRNA signatures correlate with tumor progression, metastatic potential, and treatment response, further indicating their utility in clinical applications (30).

Recent studies have explored the potential of saliva as a diagnostic medium for cancer detection (31). Cancer patients exhibit distinct miRNA profiles in saliva, which can be leveraged for non-invasive diagnosis (31). Exosomes released from cancer cells circulate through the bloodstream and can reach the salivary glands, where they are transferred to saliva via membrane fusion or endocytosis (32). This process enables the detection of cancer-derived miRNAs in saliva, presenting a highly convenient and painless alternative to traditional biopsy methods. Furthermore, given the anatomical proximity of the esophagus to the oral cavity, it is hypothesized that miRNAs released from esophageal cancer cells may directly enter the oral cavity, particularly in patients with GERD. This suggests that salivary miRNA profiles may serve as potential biomarkers for esophageal cancer diagnosis. Additionally, saliva collection is simple, cost-effective, and non-invasive, making it highly suitable for large-scale screening programs (33). The development of standardized protocols for saliva-based miRNA analysis could significantly enhance early cancer detection and improve clinical outcomes.

This meta-analysis aims to evaluate the diagnostic utility of circulating miRNAs in both blood and saliva samples of esophageal cancer patients. By systematically analyzing existing studies, we seek to determine whether miRNAs can serve as reliable liquid biopsy biomarkers for the diagnosis of esophageal cancer, thereby addressing the critical need for non-invasive and patient-friendly diagnostic tools. Furthermore, our study aims to compare the diagnostic accuracy of blood-derived versus saliva-derived miRNAs to identify the most effective method for early esophageal cancer detection. By highlighting the potential of liquid biopsy, we aspire to contribute to the advancement of personalized cancer diagnostics and the implementation of minimally invasive screening approaches in clinical practice.

## 2 Materials and methods

### 2.1 Search strategy

A systematic literature search was conducted using PubMed, Web of Science, and Scopus to identify studies on microRNA (miRNA) expression in esophageal cancer patients using liquid biopsy techniques. The search terms included “esophageal cancer”, “microRNA (or miRNA)”, “liquid biopsy”, and “diagnosis”. Studies published in English within the last 15 years were considered to ensure relevance. Only peer-reviewed articles were included to maintain scientific rigor and reliability. The study selection followed PRISMA guidelines, with a PRISMA flow diagram, illustrating the identification, screening, eligibility, and inclusion process (34). This structured approach ensured transparency and minimized selection bias, allowing for a reproducible methodology. The initial search results were screened based on titles and abstracts, followed by full-text reviews to assess eligibility.

### 2.2 Eligibility criteria

Duplicate records were removed to avoid redundancy. Original research articles focusing on esophageal cancer patients were included, while studies using cell lines, animal models, case reports, reviews, editorials, and conference abstracts were excluded to maintain clinical applicability and relevance. Studies investigating miRNAs in plasma, serum, or saliva samples were considered. Eligible studies had to report diagnostic test performance data, including true positives (TP), true negatives (TN), false positives (FP), and false negatives (FN). If these values were not explicitly provided, studies were included only if sensitivity and specificity data allowed their calculation. Studies lacking sufficient data were excluded to ensure comprehensive diagnostic evaluation. Studies were required to employ validated miRNA detection methods such as polymerase chain reaction (PCR) or next-generation sequencing (NGS). Studies utilizing unverified, low-sensitivity techniques or lacking robust validation were excluded to ensure comparability and reliability of the findings.

### 2.3 Data extraction and quality assessment

Data extraction was conducted systematically. Extracted data included the first author, year of publication, investigated miRNAs, number of patients and controls, sample type, and detection method. TP, TN, FP, and FN values were collected or calculated where necessary for standardized diagnostic accuracy assessment. To ensure methodological robustness, study quality was assessed using the QUADAS-2 (Quality Assessment of Diagnostic Accuracy Studies-2) tool (35). This assessment covered four domains: patient selection, index test, reference standard, and flow and timing. Each domain was evaluated for risk of bias and applicability concerns. Discrepancies in assessment were resolved through consensus discussions among reviewers. Results of the quality assessment were visualized using RevMan (v.5.4), ensuring clarity in study evaluation.

### 2.4 Statistical analysis

Statistical analyses were performed using Stata (v.18). Pooled sensitivity, specificity, diagnostic likelihood ratios (DLR positive and negative), diagnostic score (DS), and diagnostic odds ratio (DOR) were calculated. Summary receiver operating characteristic (SROC) curves and the area under the curve (AUC) were utilized to evaluate diagnostic accuracy across studies, allowing for an overall estimation of the diagnostic performance of circulating miRNAs in esophageal cancer detection. Statistical significance was determined at a p-value of <0.05. Heterogeneity among studies was assessed using Cochran’s Q test and the I^2^ statistic, with an I^2^ value above 50% indicating substantial heterogeneity. Due to observed heterogeneity, a random-effects model was applied to account for variations among included studies and to ensure robust meta-analytical conclusions. Publication bias was assessed using Deeks’ funnel plot asymmetry test, with a p-value of <0.05 indicating significant bias. By employing a rigorous methodology that includes a structured search strategy, strict eligibility criteria, thorough quality assessment, and comprehensive statistical analyses, this meta-analysis provides valuable insights into the role of circulating miRNAs in liquid biopsy for esophageal cancer diagnosis.

## 3 Results

### 3.1 Study selection

**Figure 1:**
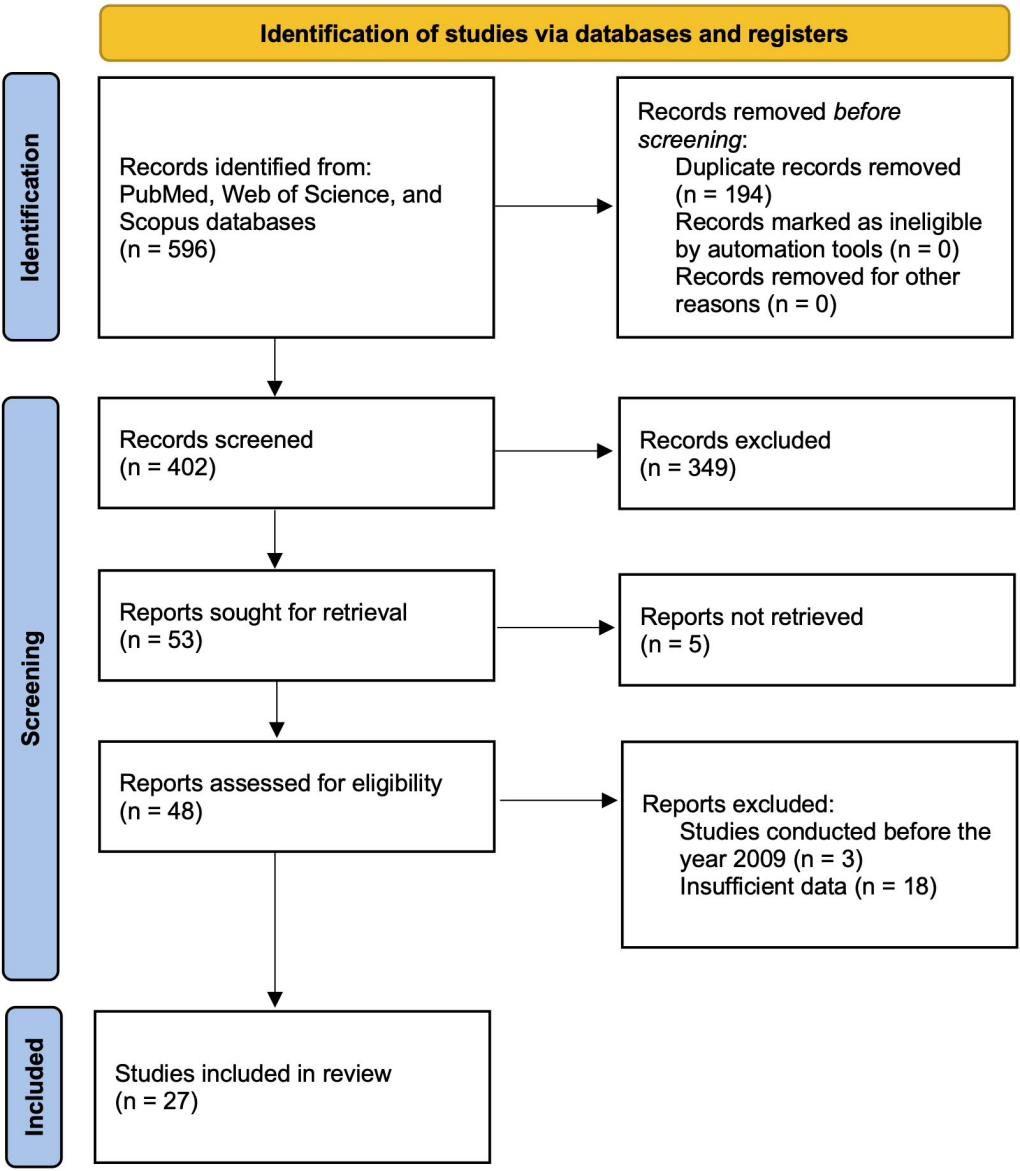
PRISMA flow diagram illustrating the study selection process.

A comprehensive literature search was conducted using PubMed, Web of Science, and Scopus, which initially identified 596 articles. Following the removal of 194 duplicate records, 402 articles remained for screening. The titles and abstracts of these articles were examined in detail, leading to the exclusion of 349 studies that did not meet the inclusion criteria. Subsequently, full texts of the remaining 53 articles were retrieved, but five were inaccessible due to limitations in database access. A further eligibility assessment resulted in the exclusion of three articles because they had been published more than 15 years ago, while 18 were excluded due to insufficient data reporting. Ultimately, 27 articles met all criteria and were included in this meta-analysis, with the selection process summarized in the PRISMA flow diagram (**Figure 1**).

Among the included articles, several contained multiple sub-studies investigating different miRNAs. Given the methodological distinctions in these sub-studies, each was treated as an independent study within the meta-analysis. As a result, the final dataset comprised 47 sub-studies, which are presented in **Table 1**. To ensure clarity in referencing, when multiple sub-studies originated from the same publication, an alphabetical designation was appended to the publication year, as in Zhang et al. (2010a).

**Table 1:** Characteristics of the studies included in the meta-analysis.

Author, year	miRNA	Patients	Controls	TP	FP	FN	TN	Sample	Ref
Zhang et al. (2010a)	miRNA127-3p	149	100	117	13	32	87	Serum	(42)
Zhang et al. (2010b)	miRNA-133a	149	100	97	17	52	83	Serum	(42)
Zhang et al. (2010c)	miRNA-223	149	100	124	17	25	83	Serum	(42)
Zhang et al. (2010d)	miRNA-148b	149	100	99	13	50	87	Serum	(42)
Zhang et al. (2010e)	miRNA-100	149	100	95	19	54	81	Serum	(42)
Zhang et al. (2010f)	miRNA-22	149	100	132	14	17	86	Serum	(42)
Zhang et al. (2010g)	miRNA-10a	149	100	121	20	28	80	Serum	(42)
Komatsu et al. (2011)	miRNA-21, -375	50	20	44	6	6	14	Plasma	(43)
Zhang et al. (2011)	miRNA-31	201	202	174	36	27	166	Serum	(44)
Kurashige et al. (2012)	miRNA-21	71	39	33	0	38	39	Serum	(45)
Wang et al. (2012)	miRNA-21	31	39	22	12	9	27	Serum	(46)
Xie et al. (2012)	miRNA-21	32	16	28	6	4	10	Saliva	(47)
Hirajima et al. (2013)	miRNA-18a	106	54	92	0	14	54	Plasma	(48)
Takeshita et al. (2013)	miRNA-1246	101	46	72	12	29	34	Serum	(49)
Xie et al. (2013a)	miRNA-21	39	19	35	10	4	9	Saliva	(50)
Xie et al. (2013b)	miRNA-451	39	19	33	8	6	11	Saliva	(50)
Xie et al. (2013c)	miRNA-144	39	19	36	10	3	9	Saliva	(50)
Xie et al. (2013d)	miRNA-10b	39	19	35	8	4	11	Saliva	(50)
Zhang et al. (2013)	miRNA-1322	201	201	166	37	35	164	Serum	(51)
Wu et al. (2014a)	miRNA-483-5p	63	63	50	25	13	38	Serum	(52)
Wu et al. (2014b)	miRNA-337-5p	63	63	55	14	8	49	Serum	(52)
Wu et al. (2014c)	miRNA-223	63	63	47	20	16	43	Serum	(52)
Wu et al. (2014d)	miRNA-194	63	63	55	28	8	35	Serum	(52)
Wu et al. (2014e)	miRNA-193a-3p	63	63	57	24	6	39	Serum	(52)
Wu et al. (2014f)	miRNA-100	63	63	48	22	15	41	Serum	(52)
Wu et al. (2014g)	miRNA-25	63	63	50	20	13	43	Serum	(52)
Komatsu et al. (2014)	miRNA-25	20	50	17	7	3	43	Plasma	(53)
Ye et al. (2014a)	miRNA-21	100	50	89	18	11	32	Saliva	(54)
Ye et al. (2014b)	miRNA-21	100	50	97	22	3	28	Plasma	(54)
Sun et al. (2015)	miRNA-718	120	51	83	17	37	34	Plasma	(55)
Xu et al. (2015a)	miRNA-205	50	50	35	18	15	32	Serum	(56)
Xu et al. (2015b)	miRNA-29c	50	50	34	16	16	34	Serum	(56)
Xu et al. (2015c)	miRNA-10b	50	50	38	8	12	42	Serum	(56)
He et al. (2015)	miRNA-20a	70	40	45	10	25	30	Plasma	(57)
Wang et al. (2016)	miRNA-146a	154	154	129	36	25	118	Serum	(58)
Li et al. (2016)	miRNA-506	110	40	89	5	21	35	Plasma	(59)
Dong et al. (2016a)	miRNA-216b	120	51	67	5	53	46	Plasma	(60)
Dong et al. (2016b)	miRNA-216a	120	51	96	5	24	46	Plasma	(60)
Komatsu et al. (2016)	miRNA-21	37	20	20	1	17	19	Plasma	(61)
Sharma et al. (2018)	miRNA-21	24	21	20	9	4	12	Blood	(62)
Wang et al. (2018)	miRNA-21	35	32	27	5	8	27	Serum	(63)
Zhang et al. (2018)	miRNA-21	125	125	93	28	33	98	Blood	(64)
Samiei et al. (2019)	miRNA-21	34	34	26	12	8	22	Plasma	(65)
Sun et al. (2019)	miRNA-21	125	125	76	13	49	113	Plasma	(66)
Hoshino et al. (2020a)	miRNA-1246	101	34	72	10	29	24	Serum	(67)
Hoshino et al. (2020b)	miRNA-1246	55	39	40	12	15	27	Serum	(67)
Hoshino et al. (2021)	miRNA-1246	72	50	60	17	12	33	Saliva	(68)

### 3.2 Study characteristics

A total of 47 sub-studies with 2,396 patients were included in this meta-analysis. Of the 47 included sub-studies, 40 focused on blood-derived miRNAs, whereas 7 examined saliva-derived miRNAs. The studies varied significantly in terms of publication year, geographical location, sample size, and the specific miRNAs under investigation. This heterogeneity reflects the evolving nature of miRNA research and its growing recognition as a diagnostic biomarker. Sample sizes ranged from as small as 20 to more than 200 patients, ensuring a broad representation of different study populations. Despite this variation, all studies were deemed to have sufficient statistical power for inclusion in the meta-analysis. Most of the studies investigated a single miRNA, though one study (Komatsu et al., 2011) analyzed multiple miRNAs in a comprehensive manner. A detailed breakdown of study characteristics, including sample size and the specific miRNAs analyzed, is available in **Table 1**.

### 3.3 Quality assessment

**Figure 2:**
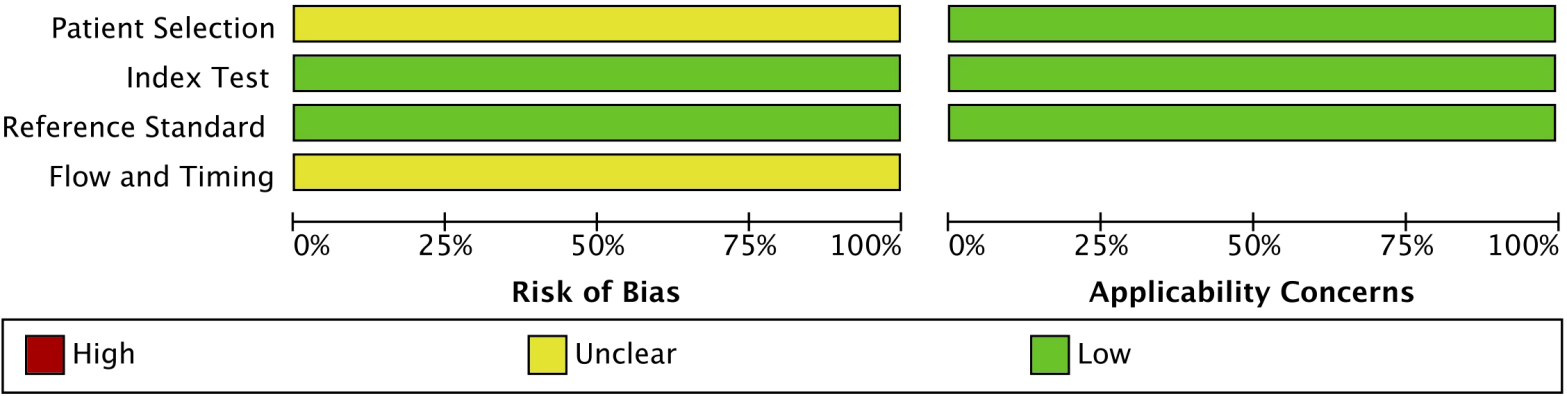
Quality assessment of the included studies using the quality assessment of diagnostic accuracy studies 2 (QUADAS-2) tool.

The quality of the included studies was systematically assessed using the QUADAS-2 tool (**Figure 2**). The evaluation indicated that the risk of bias in patient selection remained unclear due to potential selection biases in some studies. However, the index test, reference standard, and flow and timing components were found to have a low risk of bias, indicating that most studies employed rigorous diagnostic methodologies. Similarly, concerns regarding the applicability of the studies were also noted as unclear for patient selection, while the index test and reference standard were assessed as having low concerns. The QUADAS-2 assessment confirmed that the studies included in this meta-analysis were of acceptable quality, supporting the reliability and robustness of the findings. These results indicate the need for standardization in miRNA research to improve the comparability of future studies.

### 3.4 Meta-analysis

**Figure 3:**
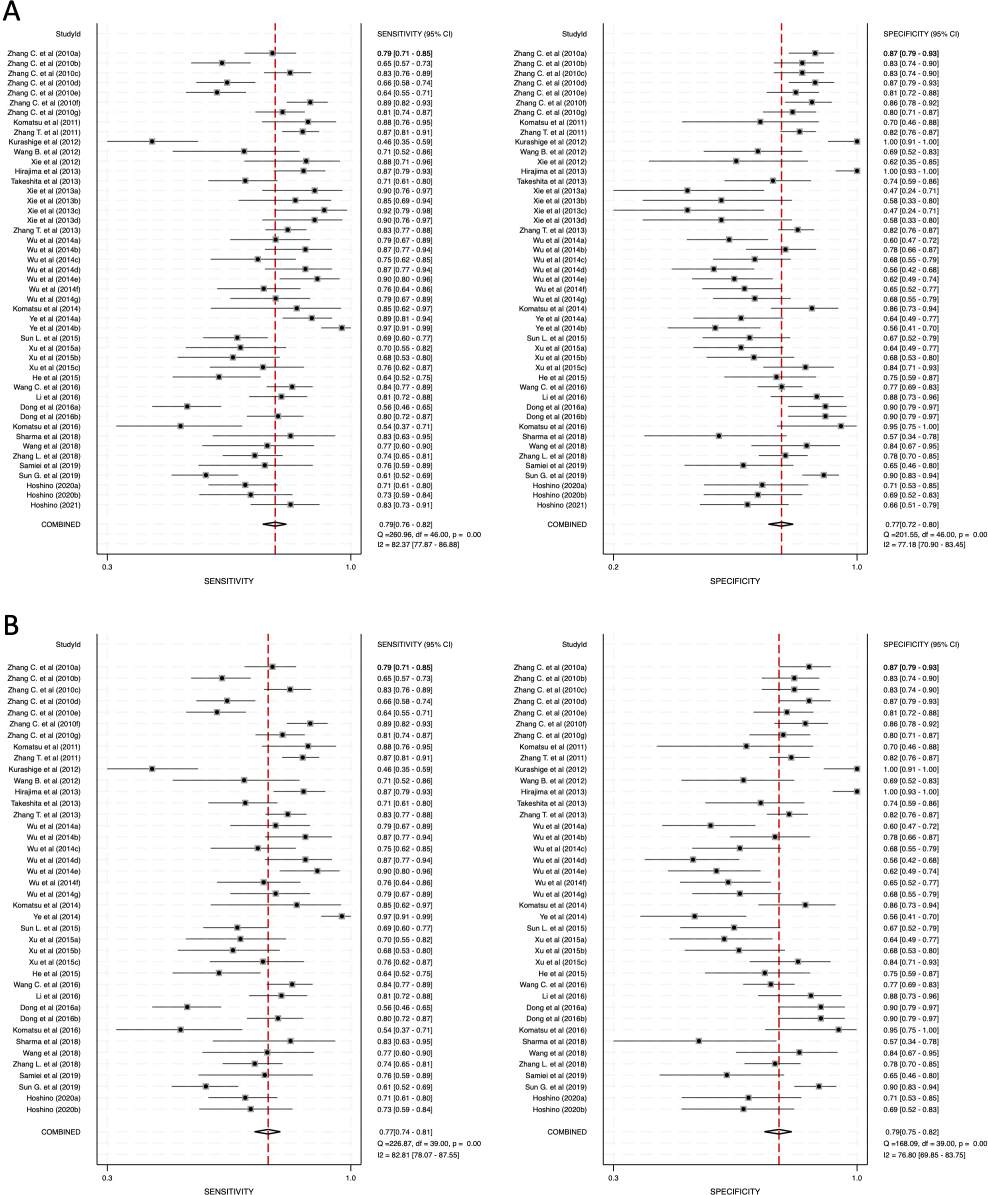
Pooled sensitivity and specificity for circulating miRNA-based diagnostics of esophageal cancer. **(A)** Combined blood- and saliva-derived miRNAs (Sensitivity: 0.79, Specificity: 0.77). **(B)** Blood-derived miRNAs alone (Sensitivity: 0.77, Specificity: 0.79).

The meta-analysis was conducted across three primary categories: 1) combined blood- and saliva-derived miRNAs, 2) blood-derived miRNAs, and 3) saliva-derived miRNAs. The pooled sensitivity for combined blood- and saliva-derived miRNAs was 0.79 (95% CI: 0.76–0.82), while blood-derived miRNAs demonstrated a sensitivity of 0.77 (95% CI: 0.74–0.81) (**Figures 3A, B**). Saliva-derived miRNAs, however, exhibited the highest sensitivity at 0.88 (95% CI: 0.84–0.91) (**Supplementary Figure 1**). These results indicate that saliva-derived miRNAs are particularly effective in identifying true positive cases, although their specificity may be comparatively lower.

The specificity analysis revealed that combined blood- and saliva-derived miRNAs had a specificity of 0.77 (95% CI: 0.72–0.80), while blood-derived miRNAs demonstrated a slightly higher specificity of 0.79 (95% CI: 0.75–0.82) (**Figures 3A, B**). Saliva-derived miRNAs, however, showed a lower specificity of 0.60 (95% CI: 0.53–0.67) (**Supplementary Figure 1**). These findings suggest that while saliva-derived miRNAs are highly sensitive, they may be associated with a higher false-positive rate compared to blood-derived miRNAs.

**Figure 4:**
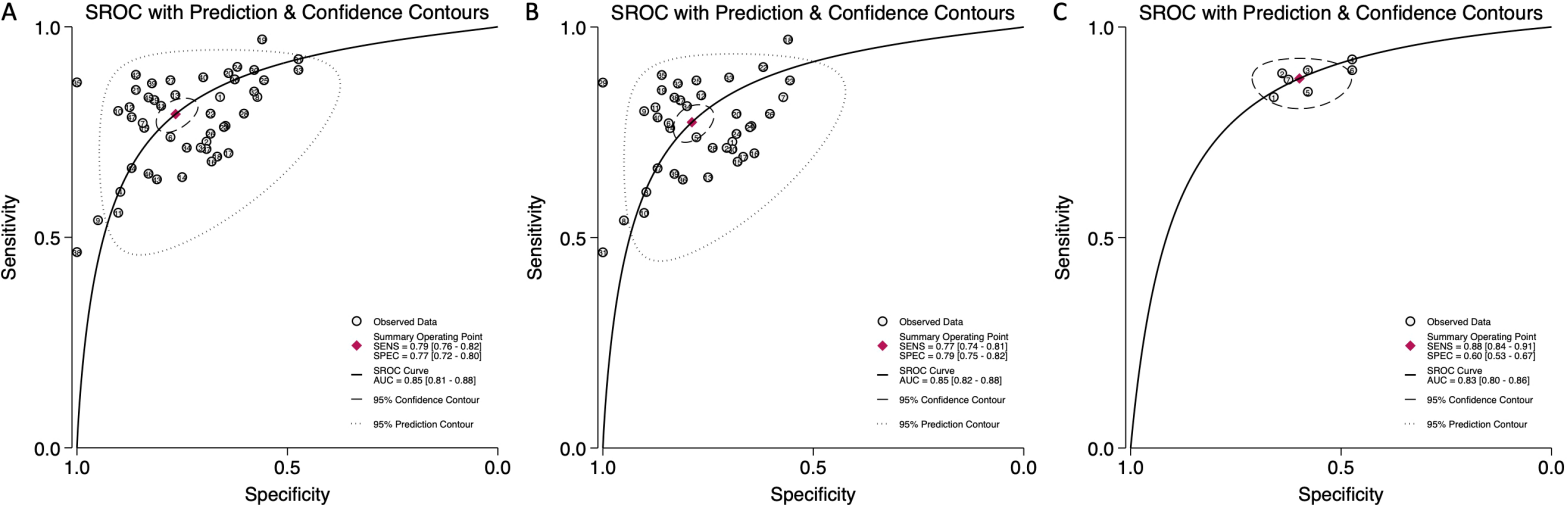
Summary receiver operating characteristic (SROC) curve analysis for circulating miRNA-based esophageal cancer diagnostics. **(A)** Combined blood- and saliva-derived miRNAs (AUC: 0.85, 95% CI: 0.81–0.88). **(B)** Blood-derived miRNAs alone (AUC: 0.85, 95% CI: 0.82–0.88). **(C)** Saliva-derived miRNAs alone (AUC: 0.83, 95% CI: 0.80–0.86).

The Summary Receiver Operating Characteristic (SROC) curves illustrated strong diagnostic performance across all categories, with the area under the curve (AUC) values calculated as 0.85 (95% CI: 0.81–0.88) for combined blood- and saliva-derived miRNAs, 0.85 (95% CI: 0.82–0.88) for blood-derived miRNAs, and 0.83 (95% CI: 0.80–0.86) for saliva-derived miRNAs (**Figure 4**). These values demonstrate the high overall diagnostic accuracy of miRNA-based biomarkers.

**Figure 5:**
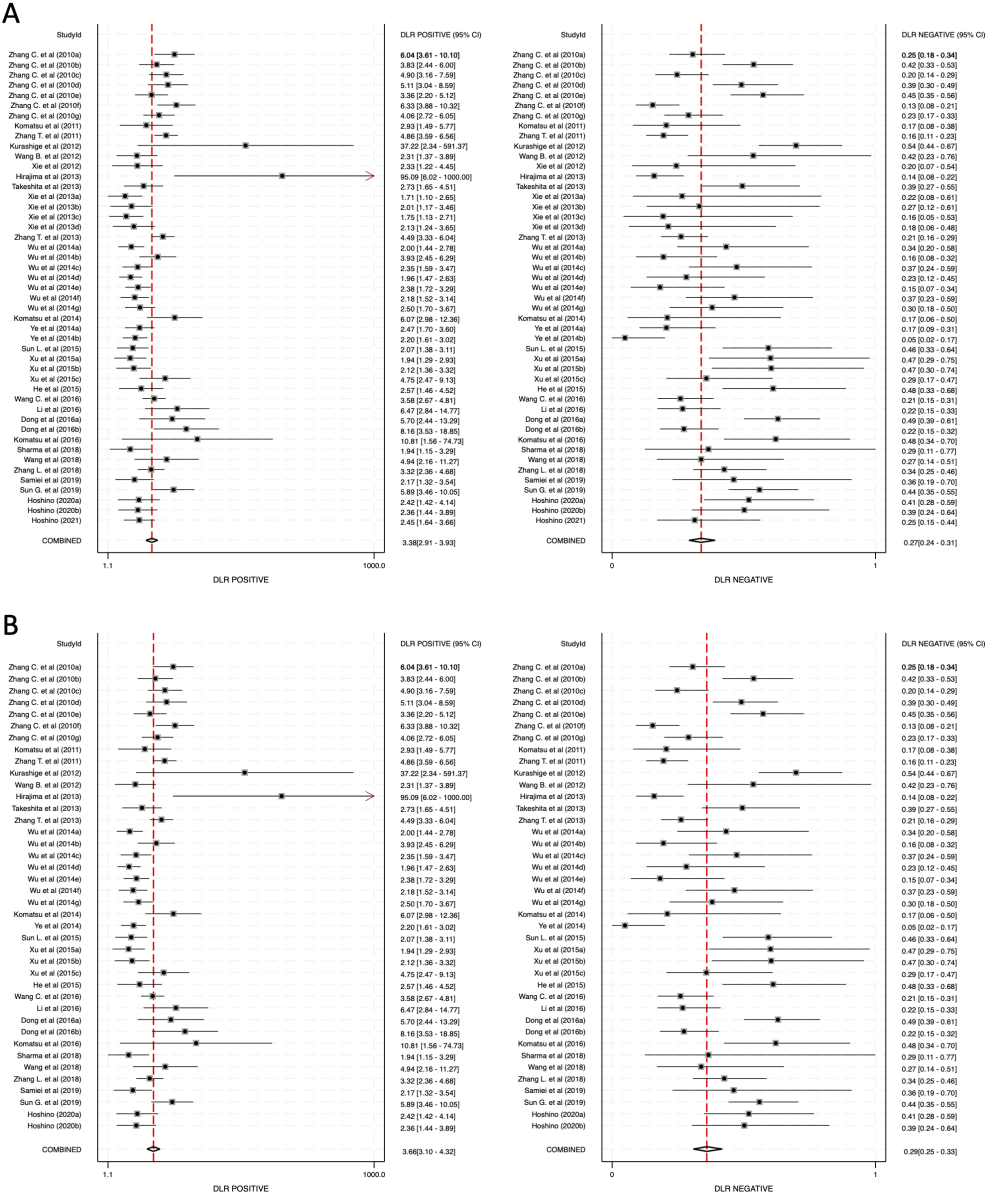
Diagnostic likelihood ratios (DLRs) for circulating miRNA-based esophageal cancer diagnostics. **(A)** Combined blood- and saliva-derived miRNAs (DLR positive: 3.38, DLR negative: 0.27). **(B)** Blood-derived miRNAs alone (DLR positive: 3.66, DLR negative: 0.29).

Further supporting these results, the diagnostic likelihood ratio positive (DLR-positive) values were determined to be 3.38 (95% CI: 2.91–3.93) for combined blood- and saliva-derived miRNAs, 3.66 (95% CI: 3.10–4.32) for blood-derived miRNAs, and 2.19 (95% CI: 1.83–2.61) for saliva-derived miRNAs (**Figures 5A, B**; **Supplementary Figure 2**). The corresponding negative diagnostic likelihood ratio (DLR-negative) values were 0.27 (95% CI: 0.24–0.31) for combined blood- and saliva-derived miRNAs, 0.29 (95% CI: 0.25–0.33) for blood-derived miRNAs, and 0.20 (95% CI: 0.15–0.28) for saliva-derived miRNAs (**Figures 5A, B**; **Supplementary Figure 2**). These findings indicate that while blood-derived miRNAs are superior in confirming a disease presence when a test result is positive, saliva-derived miRNAs are more reliable in ruling out disease when a test result is negative.

**Figure 6:**
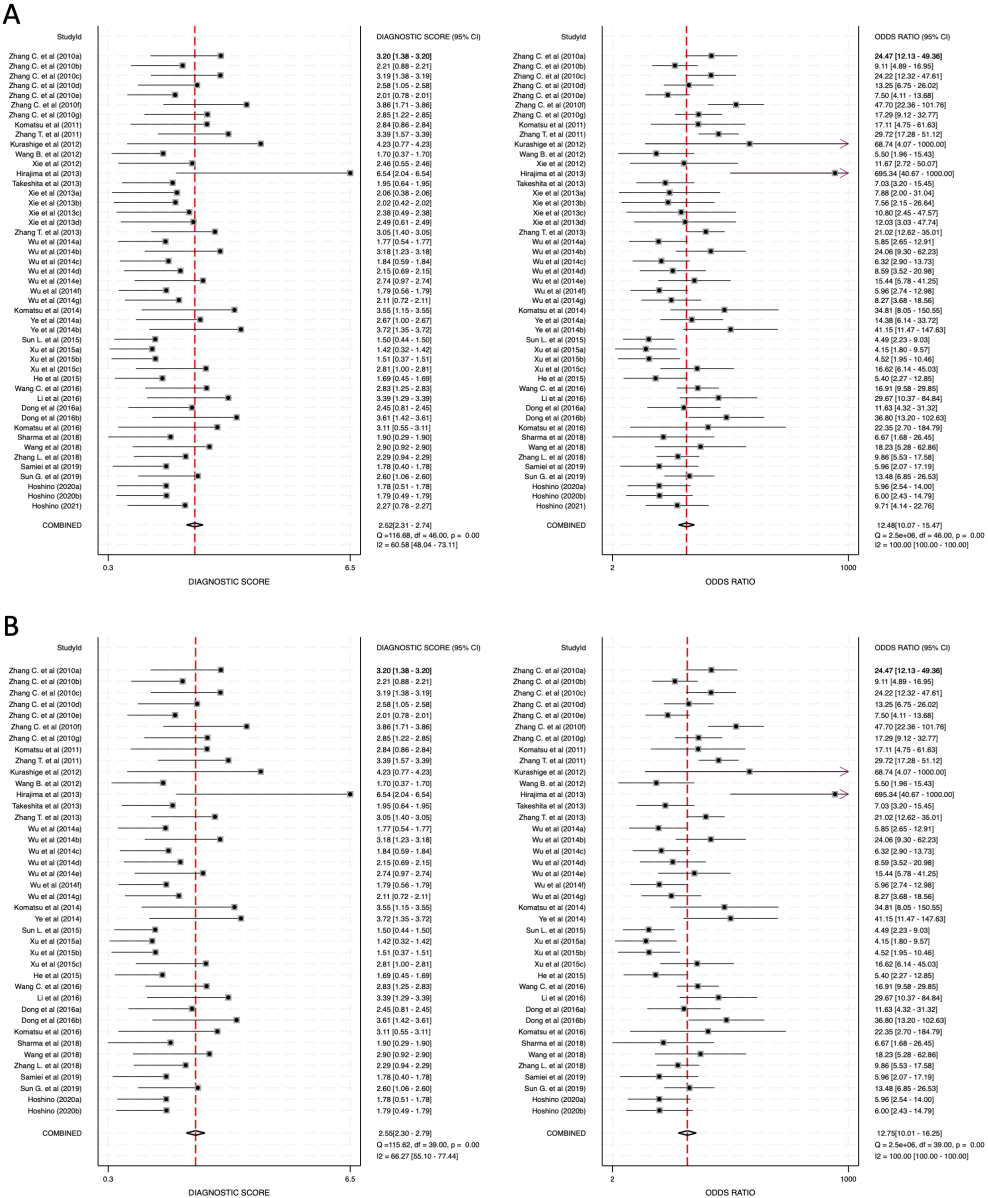
Diagnostic score (DS) and diagnostic odds ratio (DOR) for circulating miRNA-based esophageal cancer diagnostics. **(A)** Combined blood- and saliva-derived miRNAs (DS: 2.52, DOR: 12.48). **(B)** Blood-derived miRNAs alone (DS: 2.55, DOR: 12.75).

The diagnostic odds ratio (DOR) values also reinforced the strength of these findings, with DOR values of 12.48 (95% CI: 10.07–15.47) for combined blood- and saliva-derived miRNAs, 12.75 (95% CI: 10.01–16.25) for blood-derived miRNAs, and 10.73 (95% CI: 6.99–16.45) for saliva-derived miRNAs (**Figures 6A, B**; **Supplementary Figure 3**). These results suggest that miRNAs hold substantial potential as diagnostic biomarkers.

### 3.5 Publication bias

**Figure 7:**
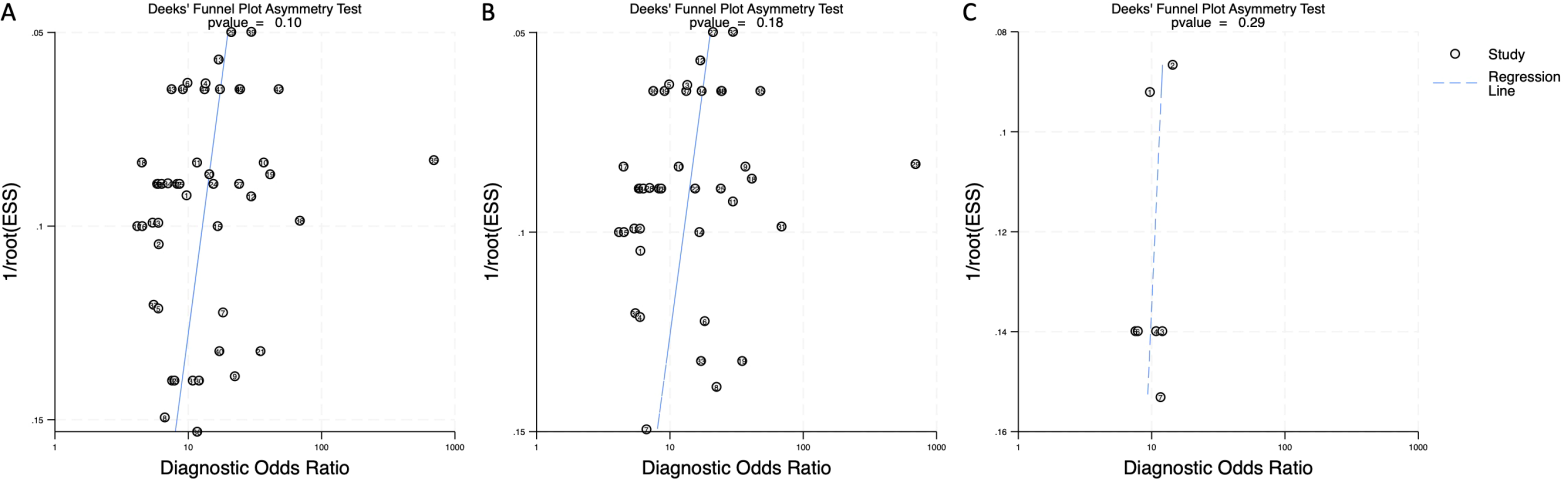
Deeks’ funnel plot assessing publication bias in circulating miRNA-based esophageal cancer diagnostics. **(A)** Combined blood- and saliva-derived miRNAs (p = 0.10). **(B)** Blood-derived miRNAs alone (p = 0.18). **(C)** Saliva-derived miRNAs alone (p = 0.29).

To assess the potential influence of publication bias, Deeks’ funnel plot asymmetry test was performed. The resulting p-values were 0.10 for combined blood- and saliva-derived miRNAs, 0.18 for blood-derived miRNAs, and 0.29 for saliva-derived miRNAs (**Figure 7**). Since all values exceeded 0.05, no significant evidence of publication bias was detected, affirming the robustness of the meta-analysis findings.

## 4 Discussion

This meta-analysis is the first study to directly compare the diagnostic performance of blood-derived miRNAs and saliva-derived miRNAs, while also evaluating the utility of combining both sources for improved diagnostic accuracy. The results demonstrated that blood-derived miRNAs exhibit a balanced diagnostic profile with a sensitivity of 0.77 and specificity of 0.79, while saliva-derived miRNAs showed higher sensitivity of 0.88 but lower specificity of 0.60. The combined analysis of both blood- and saliva-derived miRNAs achieved an area under the curve (AUC) of 0.85, indicating strong overall diagnostic accuracy. Furthermore, the positive diagnostic likelihood ratio were highest for blood-derived miRNAs (DLR-positive = 3.66), whereas the negative diagnostic likelihood ratio was lowest for saliva-derived miRNAs (DLR-negative = 0.20), emphasizing their potential in ruling out disease when test results are negative. These findings suggest that while blood-derived miRNAs are well-suited for confirmatory diagnostics due to their higher specificity, the inclusion of saliva-derived miRNAs can enhance early disease detection through increased sensitivity. By systematically assessing the strengths and limitations of each biofluid, this study provides novel insights into the complementary role of blood- and saliva-derived miRNAs in non-invasive disease detection.

The use of blood-derived miRNAs in liquid biopsy presents a significant improvement over traditional invasive procedures such as tissue biopsies. Blood sampling is less painful, reduces patient discomfort, and allows for real-time disease monitoring through repeated testing. Additionally, liquid biopsy can detect biomarkers before visible pathological changes occur, enabling earlier intervention and improving patient outcomes. The high specificity of blood-derived miRNAs (specificity = 0.79) also suggests their suitability for confirmatory diagnostics, minimizing the risk of false-positive diagnoses that can lead to unnecessary treatments. Furthermore, the diagnostic odds ratio of blood-derived miRNAs (DOR = 12.75) indicates strong diagnostic power, reinforcing their utility in distinguishing disease presence with greater accuracy. The superior positive diagnostic likelihood ratio (DLR-positive = 3.66) further supports their role in enhancing diagnostic certainty.

While blood-derived miRNAs alone offer significant diagnostic advantages, combining both blood- and saliva-derived miRNAs enhances overall performance. The meta-analysis showed that combined blood- and saliva-derived miRNAs achieved an area under the curve (AUC) of 0.85, demonstrating superior diagnostic accuracy. By integrating saliva-derived miRNAs, which exhibit higher sensitivity of 0.88, the combined approach enhances the ability to identify true positive cases, making it particularly useful for early disease screening. Moreover, the combined use of blood and saliva samples allows for a more comprehensive biomarker profile, potentially capturing disease-specific miRNAs that may not be equally expressed in both fluids. This multimodal approach can improve diagnostic robustness, especially in cases where a single biofluid might not provide sufficient information for conclusive results.

Although blood-derived miRNAs remain central to liquid biopsy research, saliva-derived miRNAs offer additional benefits. Saliva collection is entirely non-invasive, making it highly accessible for large-scale screenings (33). Despite their lower specificity of 0.60, saliva-derived miRNAs serve as an effective initial screening tool, reducing the need for more invasive tests unless necessary. The negative diagnostic likelihood ratio of saliva-derived miRNAs (DLR-negative = 0.20) also indicates their strong ability to rule out disease when test results are negative. Given its low DLR-negative value, saliva-derived miRNAs merit particular attention as a frontline screening modality. In practical terms, this means that a negative test result using salivary miRNAs substantially lowers the likelihood of esophageal cancer, making it an effective triage tool in asymptomatic or at-risk populations. This strong “rule-out” capability highlights saliva-based testing as a clinically valuable strategy to reduce false negatives, minimize unnecessary endoscopies, and enable broader community-based screening programs, especially in settings where access to invasive diagnostic tools is limited. This suggests that integrating saliva-derived miRNAs could improve overall diagnostic efficiency when used in conjunction with blood-derived miRNAs.

One notable finding of this meta-analysis is the high sensitivity of saliva-derived miRNAs (sensitivity = 0.88). This can be partially explained by the anatomical proximity between the oral cavity and the esophagus. Food and drink pass through the esophagus to the stomach, but this movement is not strictly one-way. Even in healthy individuals, gastric contents can reflux into the oral cavity during belching, and in cases of vomiting, stomach contents return to the mouth. Studies have shown that certain bacterial populations are shared between the oral cavity and the stomach, with oral microbiota influencing gastric conditions and vice versa (36–38). For example, in gastric cancer patients, bacteria such as Fusobacterium and Butyrivibrio are found in both the oral cavity and gastric acid (39). Post-gastrectomy, these bacterial populations decrease in the oral cavity, supporting the idea that gastric microbiota can return to the oral cavity (39). In esophageal cancer patients, symptoms such as difficulty swallowing and nausea often lead to increased esophageal pressure, forcing contents back toward the oral cavity (40). Additionally, many esophageal cancer patients suffer from gastroesophageal reflux disease (GERD), where stomach acid frequently reaches the throat, particularly during sleep (41). This reverse pressure mechanism may allow miRNAs released from esophageal cancer cells to reach the oral cavity, potentially explaining the enhanced detection rates of esophageal cancer using saliva-derived miRNAs. These findings highlight the potential utility of saliva-based diagnostics for esophageal cancer detection. Given the ease of sample collection and the non-invasive nature of saliva analysis, this approach could provide a practical and highly effective screening tool for early esophageal cancer detection.

Despite the promising findings, several limitations must be acknowledged. First, this meta-analysis included only 7 saliva-derived miRNA sub-studies compared to 40 blood-based studies. This discrepancy reflects the current state of available research rather than a selection bias. All saliva-derived studies that met our strict inclusion criteria, namely, clinical studies in esophageal cancer patients with validated detection methods and extractable diagnostic accuracy metrics, were included in the analysis. Therefore, while we acknowledge the numerical imbalance, we emphasize that the included saliva-based studies represent the full scope of currently eligible and high-quality literature on this topic. The limited number of such studies indicates the need for further larger, multi-center prospective studies for broader validation in this promising area.

There was notable heterogeneity among the included studies, as reflected by the I^2^ values of 65% for blood-derived miRNA sensitivity, 72% for blood-derived miRNA specificity, and similar substantial variability observed in saliva-derived miRNAs. This heterogeneity likely arises from differences in patient populations, miRNA quantification techniques, and statistical methodologies used across studies. A key contributor to the observed heterogeneity (I^2^) in our analysis is the absence of standardized protocols for miRNA detection and quantification. Studies differed in several technical aspects including RNA extraction methods (e.g., phenol-chloroform vs column-based purification), normalization controls (e.g., U6 snRNA or endogenous miRNAs), sample processing, and PCR reagents or platforms (e.g., SYBR Green vs TaqMan probes). These methodological discrepancies can significantly influence the reported sensitivity and specificity, leading to variability across studies. Additionally, the diversity of miRNAs investigated in different studies contributes to variability, as no universally accepted miRNA panel currently exists. The lack of a universally accepted panel of miRNAs for esophageal cancer diagnosis limits cross-study comparability and hinders biomarker validation. Therefore, standardization of pre-analytical and analytical workflows, including sample handling, quality control, normalization strategy, and miRNA panel composition, is crucial for enhancing reproducibility and clinical translation in future studies.

Another important consideration is the lack of standardized sample collection protocols across the included studies. None of the saliva-based studies reported collecting samples at a uniform clinical time point relative to disease stage or treatment, nor under controlled fasting conditions. Establishing such rigorous collection protocols would represent an ideal future direction for saliva-based miRNA research, as synchronized timing and fasting-state sampling could reduce biological variability and enhance biomarker reliability. Achieving this level of standardization would likely require a prospective diagnostic clinical trial framework with dedicated facilities, coordinated scheduling, and strong participant adherence. Although such trials have not yet been conducted in saliva diagnostics for any cancer type, they represent an important and aspirational next step toward elevating methodological consistency and advancing the clinical translation of salivary miRNA biomarkers.

A related limination concerns the control of pre-analytical variables that influence miRNA quantification. In the case of blood-derived miRNAs, most studies utilized plasma samples collected in anticoagulant-treated tubes, processed and stored according to standard protocols used in hospital clinical laboratories. This generally ensures adequate control of variables such as hemolysis, storage temperature, and handling time. In contrast, saliva-based studies lack uniformly defined pre-analytical procedures. In the field of saliva diagnostics, it is common practice for patients to spit directly into 50 mL tubes, with samples then stored in deep freezers, but details regarding handling time or preservation conditions are rarely standardized. Notably, none of the saliva-based studies included in our meta-analysis provided explicit information on pre-analytical controls. As such, these variables were not incorporated into our eligibility criteria. While this reflects a broader limitation in the current literature, we believe that future studies should aim to establish standardized pre-analytical protocols, particularly for saliva-based miRNA diagnostics, to improve reproducibility and facilitate clinical translation.

Although the use of a random-effects model mitigates some of these issues, standardization of experimental procedures, including consensus on miRNA targets and analytical methods, remains crucial for improving reproducibility in future research. Future studies should focus on increasing sample sizes and improving representation of saliva-derived miRNAs while developing standardized miRNA panels to enhance diagnostic consistency. Standardizing quantification protocols and normalization strategies will minimize variability and improve reproducibility. Additionally, advanced computational techniques, such as machine learning, can refine biomarker selection and optimize diagnostic accuracy.

## 5 Conclusion

This meta-analysis demonstrates the strong diagnostic potential of blood-derived miRNAs, particularly in the context of liquid biopsy for esophageal cancer. Their high specificity and balanced sensitivity make them a reliable alternative to invasive diagnostic procedures, offering significant advantages in patient comfort and disease monitoring. The incorporation of saliva-derived miRNAs further enhances diagnostic accuracy by improving sensitivity and reducing false negatives. A combined blood- and saliva-derived approach holds promise for optimizing non-invasive diagnostics, with the potential to transform early disease detection and clinical decision-making.

## Data Availability

The original contributions presented in the study are included in the article/**Supplementary Material**. Further inquiries can be directed to the corresponding author.
