# Supplementary material for: Saliva and blood miRNAs as complementary biomarkers for esophageal cancer detection
Source: Front Oncol. 2026 Jan 5;15:1642705. doi: 10.3389/fonc.2025.1642705 (PMC12812666; doi:10.3389/fonc.2025.1642705)
Supplement: Supplementary file 1 [file DataSheet1.pdf]

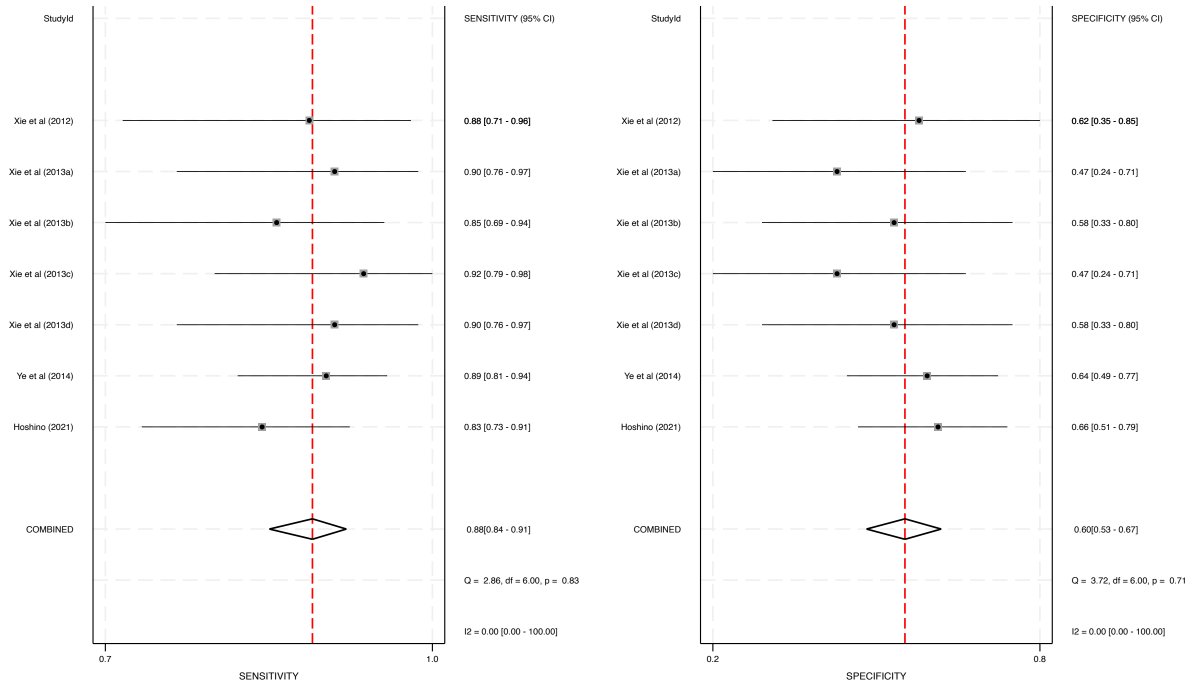

Supplementary Figure 1. Pooled sensitivity and specificity for saliva-derived miRNA alone (Sensitivity: 0.88, Specificity: 0.60).

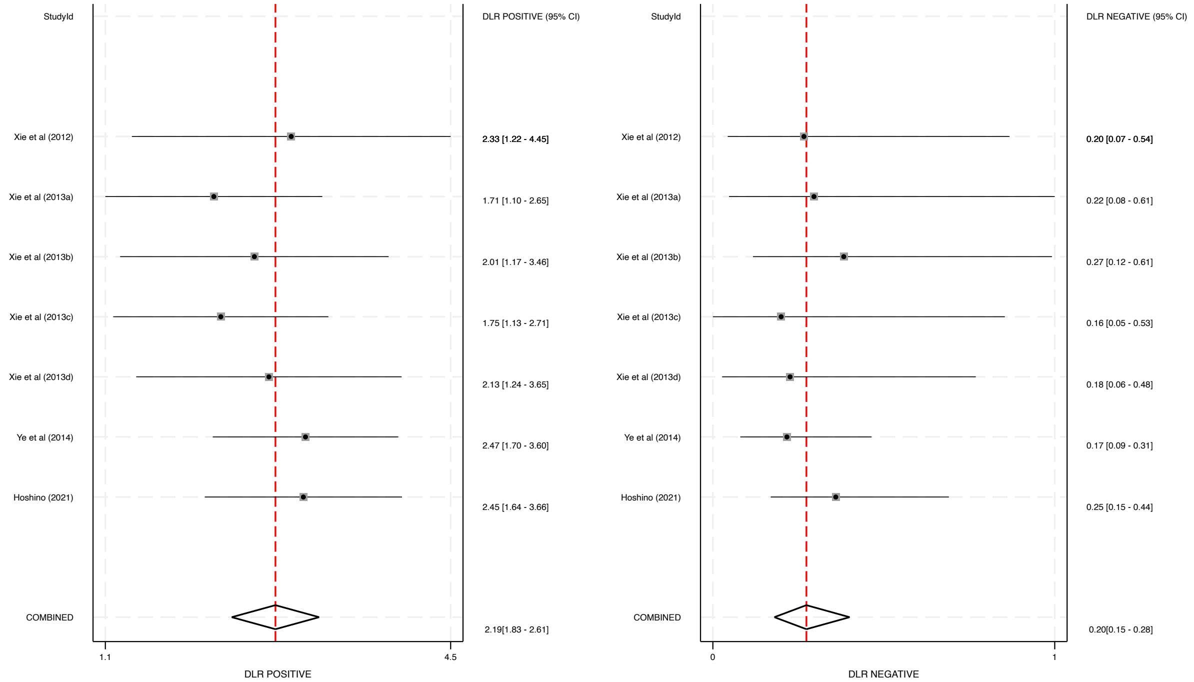

Supplementary Figure 2. Diagnostic likelihood ratios (DLRs) for saliva-derived miRNA alone (DLR positive: 2.19, DLR negative: 0.20).

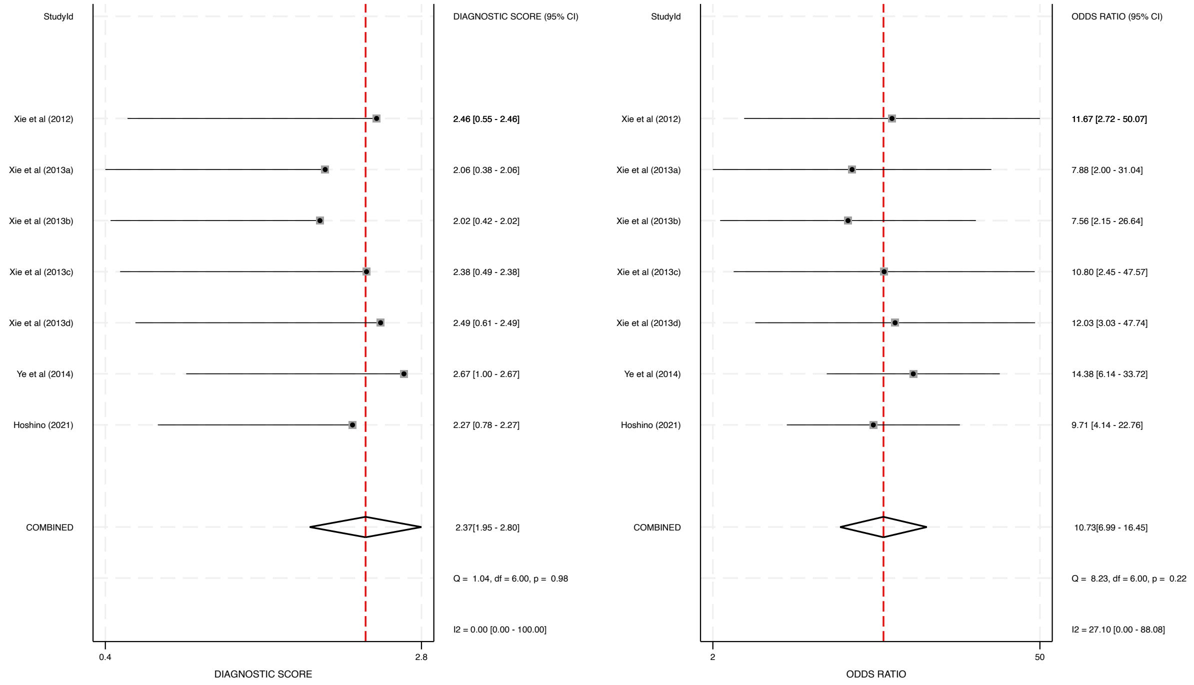

Supplementary Figure 3. Diagnostic score (DS) and diagnostic odds ratio (DOR) for saliva-derived miRNA alone (DS: 2.37, DPR: 10.73).
